# Follow-up of patients with delusional disorder in a specialized outpatient clinic over a 2-year period

**DOI:** 10.1192/j.eurpsy.2021.1371

**Published:** 2021-08-13

**Authors:** A. González-Rodríguez, A. Guàrdia, A. Alvarez Pedrero, M.V. Seeman, L. Delgado, G.F. Fucho, S. Acebillo, J.A. Monreal, D. Palao Vidal, J. Labad

**Affiliations:** 1 Mental Health, Parc Taulí University Hospital. Autonomous University of Barcelona (UAB). I3PT, Sabadell, Spain; 2 Psychiatry, University of Toronto, Toronto, Canada; 3 Mental Health, Parc Taulí University Hospital. Autonomous University of Barcelona (UAB). I3PT. CIBERSAM, Sabadell, Spain; 4 Mental Health, Hospital of Mataró. Consorci Sanitari del Maresme. CIBERSAM., Mataró, Spain

**Keywords:** Delusional disorder, psychosis, Therapy, adherence

## Abstract

**Introduction:**

In order to prevent relapse and increase medication adherence, primary care physicians and psychiatric inpatient units should consider referring patients with delusional disorder (DD) to specialized outpatient clinics for treatment and follow-up.

**Objectives:**

This poster describes a sample of DD patients referred to a specialized unit for DD and documents rates of follow-up care.

**Methods:**

Over a 2-year period, 29 individuals were consecutively referred to the Parc Tauli -Delusional Syndrome Working Group, which provides treatment and clinical care for patients with delusional disorders for a catchment area of nearly 450.000 inhabitants in Sabadell (Barcelona, Spain). Criteria for inclusion in the program are relatively flexible. Referred patients are evaluated at baseline and at 6 months following their first appointment. Treatment and case management are offered by a multidisciplinary team consisting of psychiatric, nursing, and social work personnel. Psychological interventions are also offered.

**Results:**

Of the 29 persons initially referred, 27 attended at least one scheduled appointment. Twenty-one out of the 27 patients received a confirmed diagnosis of DD (14 women,7 men), 2 suffered from schizophrenia and 4 were diagnosed with other psychiatric disorders and referred to other programs: primary care (n=2), affective program (n=1) and addictions unit (n=1). A breakdown of DD subtypes follows: persecutory (n=10,47.6%), jealous (n=4,19%), somatic (n=5,23.81%), mixed (n=2,9.5%). Three patients with DD (14.3%) were lost to follow-up. Attendance rates of the 21 DD patients: 80.4% (Women:77.67%, Men:100%).

**Conclusions:**

For a traditionally difficult-to-engage population, adherence to multidisciplinary clinic appointments was relatively high. Loss to follow-up was lower than would have been expected.

**Conflict of interest:**

AGR has received honoraria, registration for congresses and/or travel costs from Janssen, Lundbeck-Otsuka and Angelini.

